# Lecithin Organogel: A Promising Carrier for the Treatment of Skin Diseases

**DOI:** 10.1021/acsomega.3c05563

**Published:** 2024-02-20

**Authors:** Sushil Raut, Mohammed Azheruddin, Rajeev Kumar, Shivani Singh, Prabhanjan S. Giram, Deepanjan Datta

**Affiliations:** † Department of Pharmaceutics, Dr. DY Patil Institute of Pharmaceutical Sciences and Research, Pimpri, Pune, Maharashtra 411018, India; ‡ Lloyd Institute of Management and Technology, Plot No. 11, Knowledge Park-II, Greater Noida, Uttar Pradesh 201306, India; § Department of Pharmaceutical Sciences, University at Buffalo, The State University of New York, Buffalo, New York 14260, United States; ∥ Department of Pharmaceutics, Manipal College of Pharmaceutical Sciences, Manipal Academy of Higher Education, Manipal, Karnataka 576104, India

## Abstract

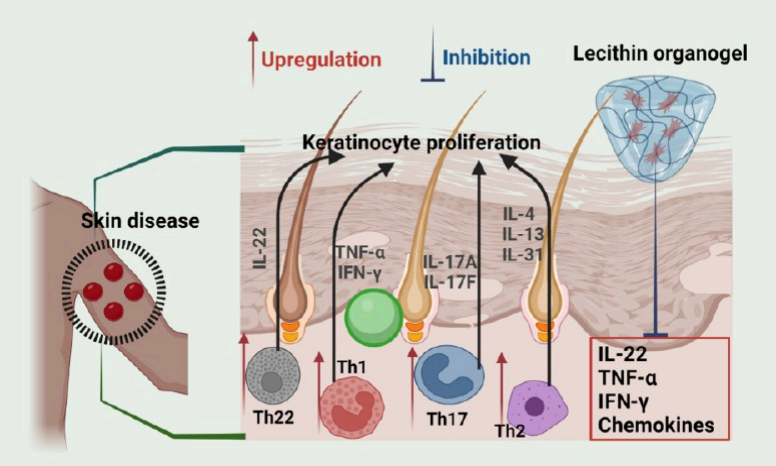

Skin is the largest organ of the human body, as it protects the body from the external environment. Nowadays, skin diseases and skin problems are more common, and millions of people are affected daily. Skin diseases are due to numerous infectious pathogens or inflammatory conditions. The increasing demand for theoretical research and practical applications has led to the rising prominence of gel as a semisolid material. To this end, organogels has been widely explored due to their unique composition, which includes organic solvents and mineral or vegetable oils, among others. Organogels can be described as semisolid systems wherein an organic liquid phase is confined within a three-dimensional framework consisting of self-assembled, cross-linked, or entangled gelator fibers. These gels have the ability to undergo significant expansion and retain substantial amounts of the liquid phase, reaching up to 99% swelling capacity. Furthermore, they respond to a range of physical and chemical stimuli, including temperature, light, pH, and mechanical deformation. Notably, due to their distinctive properties, they have aroused significant interest in a variety of practical applications. Organogels favor the significant encapsulation and enhanced permeation of hydrophobic molecules when compared with hydrogels. Accordingly, organogels are characterized into lecithin organogels, pluronic lecithin organogels, sorbitan monostearate-based organogels, and eudragit organogels, among others, based on the nature of their network and the solvent system. Lecithin organogels contain lecithin (natural and safe as a living cell component) as an organogelator. It acts as a good penetration enhancer. In this review, first we have summarized the fundamental concepts related to the elemental structure of organogels, including their various forms, distinctive features, methods of manufacture, and diverse applications. Nonetheless, this review also sheds light on the delivery of therapeutic molecules entrapped in the lecithin organogel system into deep tissue for the management of skin diseases and provides a synopsis of their clinical applications.

## 1 Introduction

**Figure 1:**
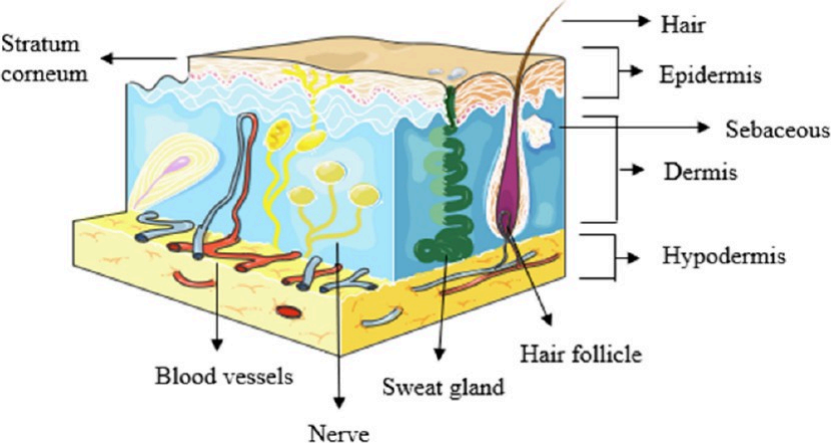
Structure of human skin.

**Figure 2:**
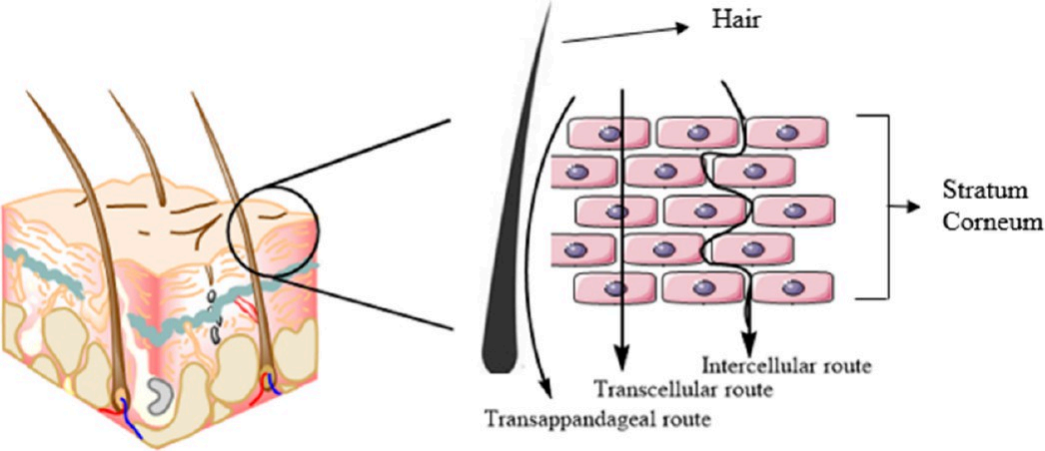
Drug diffusion transport through human skin.

The integumentary system, comprising the skin, is a vital organ within the human body due to its crucial role in protecting the individual from the external environment. The skin is comprised of three layers: the outermost epidermis layer, the inner dermis layer, and the subcutaneous fat tissue or the hypodermis layer. The differentiation between these layers is primarily based on their varying thicknesses. The epidermis has a thickness range of 50–150 μm, while the dermis has a thickness of 3–5 mm.^1^ However, the thickness of the hypodermis layer varies, and larger lymphatic and blood vessels are abundant in this layer,^2^ as illustrated in Figure 1. The epidermis lacks blood vessels, necessitating the diffusion of nutrients across the dermal–epidermal interface to sustain the viability of the epidermis. The stratum corneum (SC), which constitutes the outermost layer of the epidermis and has a thickness ranging from 15 to 20 μm, plays a crucial role in maintaining the skin’s barrier function.^3^ The concept of the SC, as a limited permeability barrier, has been schematically and mathematically represented as a two-compartment model. This model is generally represented as an impermeable matrix of cells filled with keratin embedded in a lipid matrix. Corneocytes (brick) are complex, desmosome-linked epithelial cells immersed in a lamellar structure created by intercellular lipids (mortar).^4^ The organization of this layer is reflective of the underlying skin permeation barrier.^5^ Passive diffusion is facilitated by the movement of the substances through the stratum corneum, following three probable pathways: transcellular, intercellular, and transappendageal.^6^ Transcellular or intracellular pathways favor the movement of small hydrophilic molecules, whereas the intercellular pathway favors the movement of small hydrophobic molecules. The transappendageal or shunt pathway involves the passage of molecules through sweat glands and across the hair follicles (Figure 2). Diffusion speeds are governed by lipophilicity and physical properties such as molecular weight (<500 Da), log *P* (1–3), solubility (≥1 mg/mL), melting point (<200°C), hydrogen bonding groups (<2), and permeability coefficient (>5 × 10^–3^ cm/h), among others.^7^ The diameter of lipid channels has been calculated to be 19 nm. By functioning as a barrier to external mechanical, chemical, physical, and microbiological stresses, the skin defends against infections and water loss.^8^

**Figure 3:**
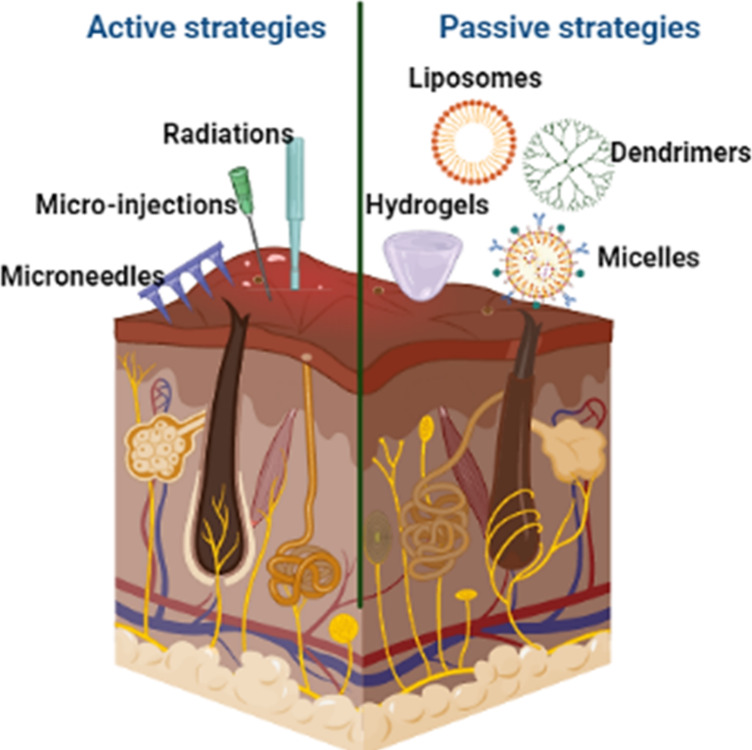
Schematic representation of drug delivery strategies.

Over the decades, various approaches have been implemented, including passive and active strategies, for the enhancement of drug retention or permeation within or across the skin (Figure 3). These strategies are noninvasive or minimally invasive in nature. Application of semisolid dosages including creams, lotions, ointments hydrogels, emulgels, bigels, and organogels, among others, has shown desirable effectiveness without any inflammation or erythema to the skin.^9−12^ However, the diffusion of the drugs from these formulations is believed to be a slower process into the system, followed by higher retention in the stratum corneum or viable epidermis (VE). The advanced drug delivery approaches include the application of permeation enhancers, nanocarriers, iontophoresis, ultrasound techniques, microneedles, medicated adhesive patches, and tape-stripping methods, which have been shown to enhance retention or skin permeation of molecules in a minimally invasive or invasive fashion.^13−16^

However, the aforementioned semisolid dosages have their own advantages and limitations. Semisolid topical dosage forms including creams, lotions, or ointments have stickiness, stability issues, and poor spreadability or permeability compared to others because of their high solid contents and the presence of mixtures of oil phases compromising their stability during storage.^17,18^ To this end, gels are a relatively newer class of dosage form that have marked applications by circumventing these limitations. Hydrogels are semisolid three-dimensional cross-linked polymeric networks that can imbibe a huge volume of water and undergo swelling or shrinkage to facilitate controlled drug-release.^19^ The high water content (70–90%) provides physical similarity to tissues and can give the hydrogels excellent biocompatibility and the ability to encapsulate hydrophilic drugs.^20,21^ However, the encapsulation of hydrophobic molecules into the aqueous network of the hydrogel is challenging.^22^ To overcome this limitation, emulgels and organogels are prepared so that the hydrophobic therapeutic moiety can be easily loaded into these gel systems.^23,24^ Emulgels generally involve the combination of both gel and emulsion. These systems also contain a gelling agent whose presence in the water phase converts an emulsion to an emulgel formulation. Various marketed formulation are available, including Voltarol, Diclomax, Dermafeet, Diclona, Cataflam, and Avindo emulgels, among others indicated for various therapeutic applications.^25^

**Figure 4:**
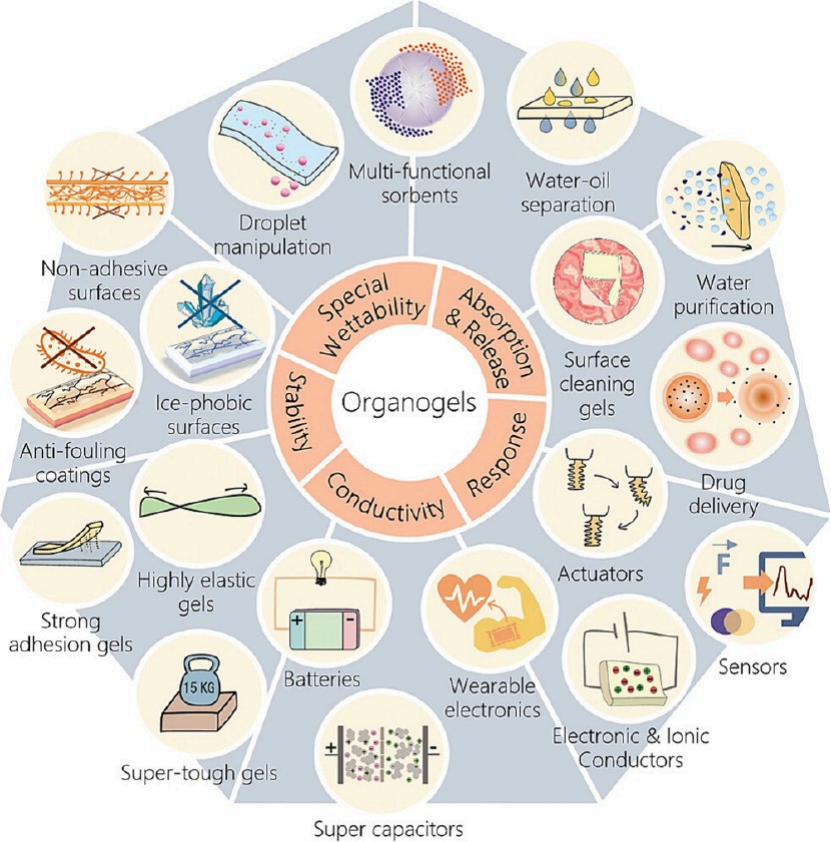
Schematic illustration of organogels with a broad range of applications in the biomedical field. Adapted and revised with permission from ref (30). Copyright 2023 Wiley.

Organogels are semisolid bicontinuous systems consisting of gelators and an apolar solvent immobilized within the available spaces of a three-dimensional network system.^26^ Recent research on organogels has shown that they have a number of desirable characteristics that could make them useful as a supplement to hydrogels in various biomaterial areas (Figure 4). Notably, these organogels can be classified into physical and chemical organogels. Physical organogels interact by noncovalent cross-linking. These are prepared by self-assembly and physical interactions of organogelator molecules without permanent cross-linking. Ideally, they exhibit viscoelastic properties.^27−29^ Limonene, sorbitan monostearate, eudragit, microemulsion–gelatin-based, and pluronic lecithin organogels are classified as physical organogels. In contrast, chemical organogels are formed through the interaction of covalent cross-links during the gelation process. In this scenario, chemical processes such as polymer chemical modification or copolymerization reactions are involved for their formation. These processes involve reactive polymers, precursors, and monomers, which trap the solvent phase and give rise to the formation of organogels.^27,28^ A supramolecular organogel is considered as a chemical-based organogel. In general, achieving a successful gelation in a specific solvent requires finding the right balance between the forces that cause gelators to aggregate and the interactions between the solvent and the gelator aggregates. More precisely, organogels can be formed by dissolving an organogelator in a hot (60–80 °C) apolar phase and then chilling it to induce gelation.

**Figure 5:**
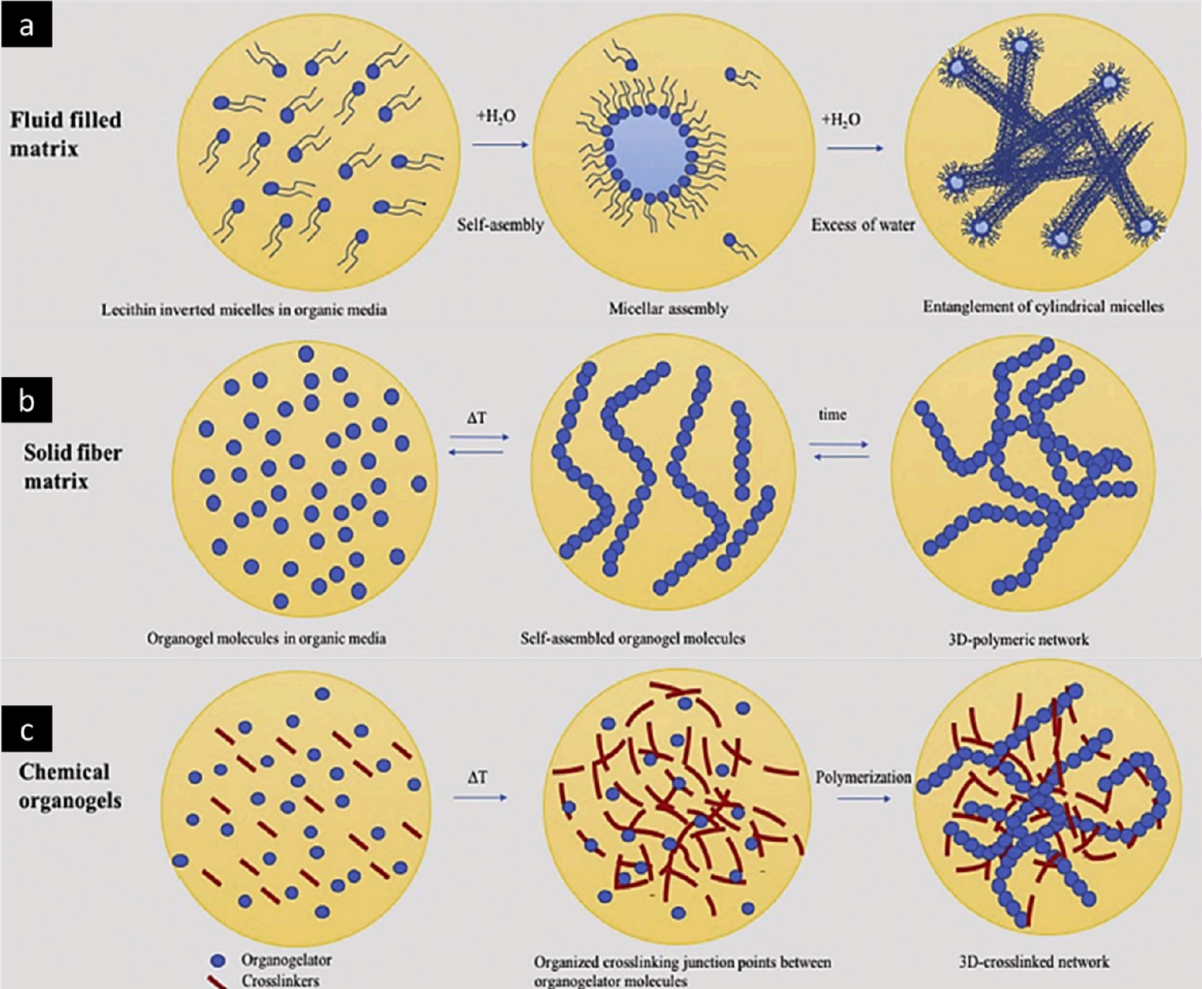
Schematic illustration of network arrangements of different types of organogels. (a) Fluid filled matrix: pluronic lecithin organogel and lecithin organogel. (b) Solid fiber matrix: limonene, sorbitan, eudragit, and microemulsion–gelatin-based organogel. (c) Chemical organogel: supramolecular organogel. Adapted and revised with permission from ref (28). Copyright 2018 Elsevier.

Lecithin molecules with both hydrophilic and hydrophobic properties spontaneously form inverted micelles when placed in an organic solvent. With an increase in concentration, cylindrical micelles are formed and intertwine to create a more intricate three-dimensional network. Formation of a dense fiber-reinforced composite is favored by incorporating organogelator molecules into an organic solvent at elevated temperatures, which results in the formation of a highly concentrated solution. Subsequently, when the temperature decreases, the molecules of the organogelator initiate the process of self-assembly, forming a network with solid-like properties and taking the form of either fibers or bundles. The self-assembly process takes place when the solubility of the organogelator declines, resulting in the formation of solid aggregates that impede phase separation. Chemical organogels resemble the inclusion of a cross-linking agent in the solution of the organogelator that initiates a chemical bonding between dissolved molecules, resulting in the formation of supramolecular aggregates. Consequently, a three-dimensional structure is formed, which permanently traps solvent molecules within the tangled, gelled system.^28^ The aforementioned discussed mechanism of organogel formation is depicted in Figure 5.

**Figure 6:**
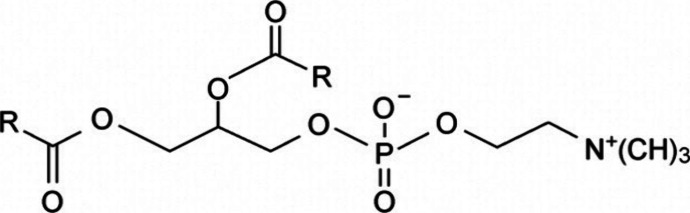
Chemical structure of lecithin.

Depending on the organogelators or active constituents, these gel systems vary in size from a few to hundreds of nanometers.^31,32^ Also, the development of these structures is driven by the arrangement of monomer units that are connected through noncovalent interactions, including van der Waals forces, hydrogen bonding, electrostatic interactions, and π–π stacking or π-stacking.^33,34^ Among the various types of organogels, lecithin organogels has been widely explored worldwide over the past few years with regard to their potential to enhance the topical and transdermal delivery of drugs.^35−41^ The chemical structure of lecithin, also known as phosphatidyl choline (a group of phospholipids, i.e., carboxylate and phosphate groups) is depicted in Figure 6 and addressed extensively in subsequent sections.

The organogels prepared can be transparent or turbid depending on the apolar solvent used.^42^ Organogelators are the main components of the organogels that produce either a solid–fiber matrix or fluid–fiber matrix when used in concentrations <15% depending on the intermolecular interactions. They have also been found to induce gelling property, even when used at very low concentrations.^43^ Cyclohexanol-derived, gemini, polymer, fatty acid, and *n*-alkane organogelators are distinct categories of organogelators, with polymer organogelators being widely employed within this classification.^44^ Isopropyl palmitate and isopropyl myristate are the commonly used apolar solvents.^27^ Unlike hydrogels, this system may or may not contain water phase. These systems have been shown to have moderate viscoelasticity, thermal reversibility, and thermal stability. Although organogels have been applied through parenteral,^45^ oral,^46^ and rectal routes,^47^ extensive research in the field of topical or transdermal application has to be explored further. To date, Diltiazem and NifeCaine (nifedipine and lidocaine) organogels are the only available marketed formulations for anal spasms and anal fissure, respectively. Taken together, in this review article, we discuss topical delivery, emphasizing the application of lecithin organogels to the skin, in order to treat cutaneous disorders or the skin-related symptoms of a general disease with the goal of limiting the drug’s pharmacological or other effects to the skin’s surface or deeper layers. Additionally, the clinical status is also addressed to provide an insight into the present situation regarding lecithin organogel cutaneous distribution.

## 2 Organogels

Organogel-based formulations have become more prevalent in recent years, possibly because their ease of preparation and long-term stability.^48^ Organogels or oleogels refer to the dispersed three-dimensional structured gels that incorporate an oil or nonpolar solvent as an external medium. They exhibit thermoreversible properties and are characterized by a network-like arrangement. The polymers forming a three-dimensional network has the ability to entrap an organic solvent, known as an organogelator.^49−51^ Organogelators are the ingredients that aid in the gelation of apolar liquids. Lecithin, sterols, cholesteryl anthraquinone derivatives, and fatty acid esters are commonly used organogelators. Organogels have the potential to enhance drug penetration across the stratum corneum due to their lipophilic nature. Fatty acids, surfactants, glycols, essential oils, and terpenes are prominent organogel components that help in the permeation process.^52^ The various advantages and limitations of organogels are depicted in Table 1.

**Table 1:** Advantages and Limitations of Organogels

sl no.	advantages	limitations
1	ease of preparation^53^	should be stored in proper conditions (4 °C and at room conditions)^54,55^
2	they are organic in character and also resist microbial contamination^56^	the organogel has greasy properties^57^
3	cost reduction due to a smaller number of ingredients^53^	less stable to extreme temperatures^58^
4	viscoelastic system with longer shelf life^59,60^	when a gel stands for some time, it often shrinks naturally and some of its liquid is pressed out, known as syneresis^61^
5	thermodynamically stable, nontoxic, and nonirritating^62^	if an impurity is present, then no gelling will occur^63^
6	both hydrophobic and hydrophilic drugs can be incorporated. organic solvents could be of natural origin, e.g., sunflower oil, mustard oil, etc.^53,64^	a raw material like lecithin is not available on a large scale^58^

### 2.1 Properties of Organogels

#### 2.1.1 Viscoelasticity

Organogels behave like a solid at low shear rates and so have an elastic characteristic.^65^ The physical interaction sites between the fiber structures reduce as the shear stress increases until the shear stress reaches a critical point that causes the rupture of the connections between the fiber structures. This rupture leads to the initiation of flow in the organogels.^66,67^

#### 2.1.2 Nonbirefringence

Birefringence refers to the optical characteristic shown by some materials that enables the transmission of polarized light as it traverses through the substance. Organogels exhibit a lack of birefringence, meaning that they impede the transmission of polarized light within their matrix. Consequently, when organogels are examined using polarized light, they exhibit a dark matrix appearance. The isotropic feature of the organogel is responsible for this phenomenon.^60,68,69^

#### 2.1.3 Thermoreversibility

The organogel matrix undergoes distortion when exposed to elevated temperatures beyond its critical temperature, resulting in a transition to a flowing state.^70^ The occurrence of thermal energy results in molecular interaction inside the organogel, leading to structural disturbances. However, as the temperature decreases, the molecular interactions likewise slow down, leading to the reversion of the organogel to its initial structure.^71^ The thermoreversibility property of the organogels encompasses the entirety of this occurrence.^72^

#### 2.1.4 Thermostability

Organogels are inherently thermostable, which can be attributed to the ability of the organogelators to self-assemble under suitable conditions to form organogels.^73^ The decrease in the total free energy helps the organogel matrix to create a low-energy thermostable system.^74,75^ At higher temperatures, the molecules within the organogel gain kinetic energy in order to mitigate any structural degradation, whereas at lower temperatures they revert back to their initial structure.^26,76^ The inherent characteristic of the organogel is accountable for its extended duration of storage, therefore rendering it an ideal carrier for the delivery of therapeutic agents.^77,78^

#### 2.1.5 Biocompatibility

Organogels were previously fabricated using incompatible components, which aroused compatibility issues. However, recent research on biocompatible components in organogels has opened up new possibilities for their usage in biological applications.^27,79,80^ To this end, lecithin organogels has been widely studied as carriers for topical administration, primarily because of their low skin irritation and inherent biocompatibility. For instance, lipid-based formulations containing highly degradable fenretinide exhibited a favorable shelf life of up to four months. These formulations showed retention of 90% of the fenretinide inside their organic structure, suggesting the use of a biocompatible substance that enhances penetration.^81^

### 2.2 Evaluation of Organogels

#### 2.2.1 Physiochemical Properties

**Figure 7:**
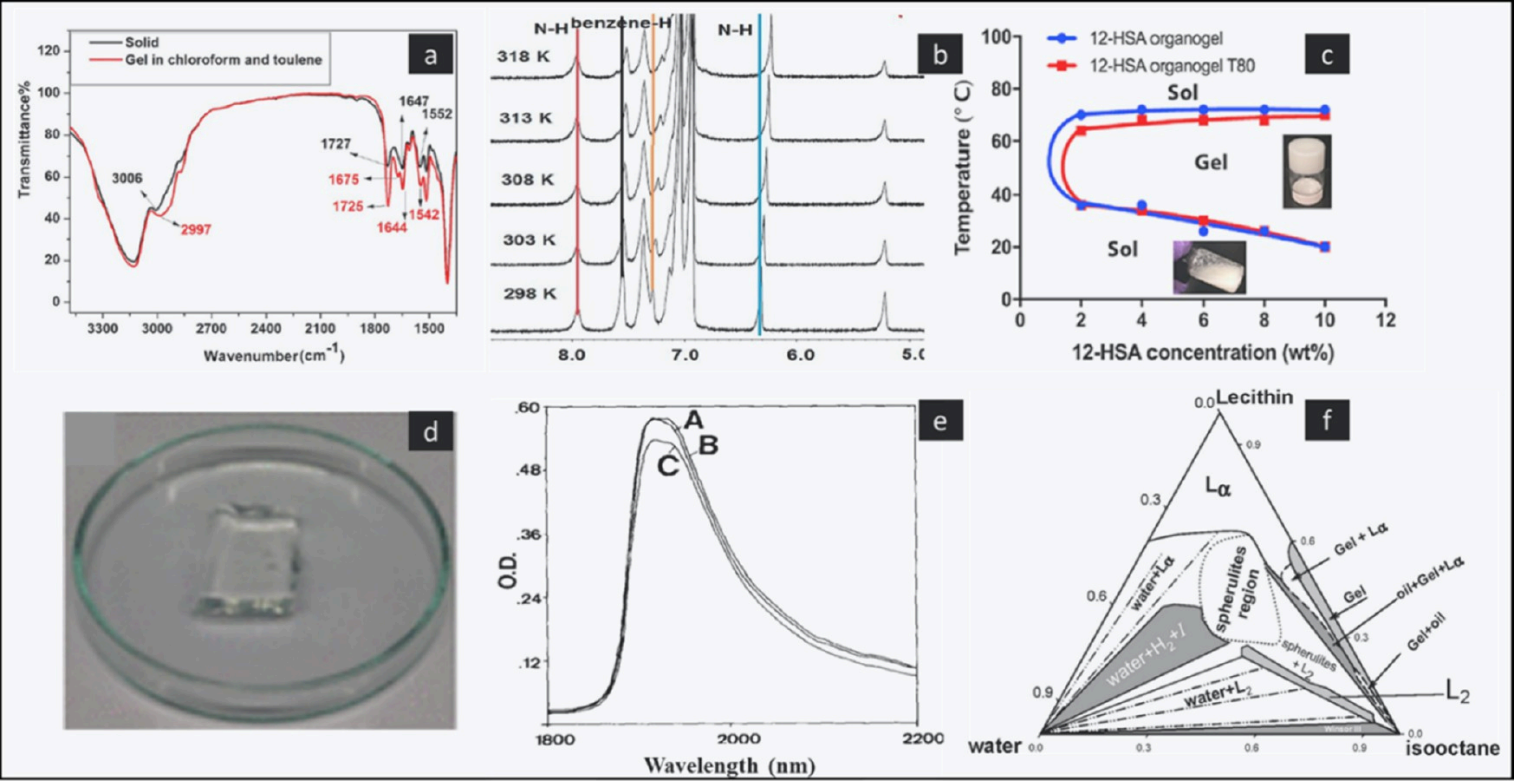
Evaluation of a fabricated organogel through (a) FT-IR and (b) NMR spectra studies indicating NH bend shift (1552–1542 cm^–1^; sol–gel state) and upfield shift of amide and aromatic protons (gel–sol state), respectively. Adapted from ref (106). Copyright 2019. (c) Temperature-dependent thermoreversible transition of a 12- hydroxystearic acid organogel. Adapted with permission from ref (107). Copyright 2020 Elsevier. (d) Image captured of a swollen nanocomposite organogel formulation. Photograph courtesy of Kaniewska from ref (96). Copyright 2024. (e) Water content determination of an organogel showing spectra at 1918 nm at day 0 (A), after 15 days (B), and after 60 days (C). Adapted with permission from ref (98). Copyright 1994 Elsevier. (f) Phase behavior of a lecithin oil–water system. Reproduced from ref (105). Copyright 2004 American Chemical Society.

The physicochemical properties of the organogels are determined by their structural characteristics. The isotopic nature and optical clarity of an organogel can be investigated using a variety of spectroscopic methods, including NMR and FT-IR spectroscopy.^82^ In the study reported by Lu et al., the self-assemble organogel of ursolic acid was characterized by NMR and FT-IR spectroscopy, which showed that the main factors that caused the aggregation and formation of the organogel were intermolecular hydrogen bonding and π–π stacking interactions (Figure 7a and b).

#### 2.2.2 Thermoreversibility (Gel to Sol and Sol to Gel Transition Test)

**Figure 8:**
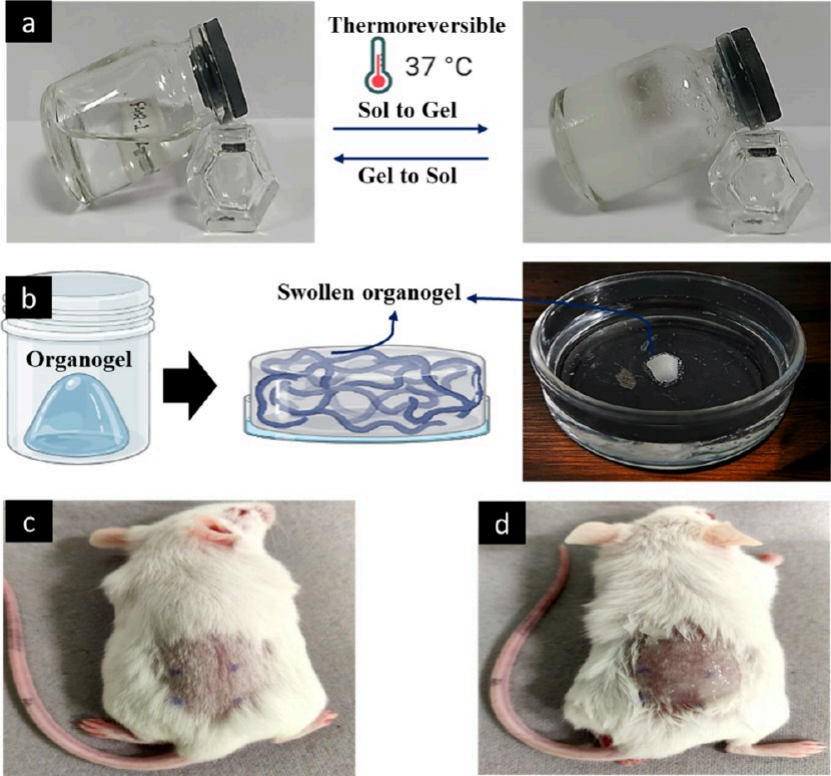
(a) Digital photographs depicting the transformation from sol to gel and vice versa. (b) Schematic and digital image of a swollen organogel formulation. Application of an organogel at (c) the 0th time point and (d) after 48 h, revealing no skin irritation or signs of erythema. Datta et al., unpublished data. Photograph courtesy of Deepanjan Datta.

The temperature of the gel–sol transition was measured by incubating a glass vial filled with organogels in a water bath at temperatures ranging from 27 to 50 °C. The temperature at which the gels began to flow was determined as the gel–sol transition when the glass vials were inverted (Figure 8a).^83^ In this process, the organogels undergo a transition in which their solid matrix-like structure is disrupted, resulting in a transition to a flowing state.^84^ This has been attributed to the disruption in the physical interactions among the gelators molecules due to the increase in the thermal energy within the organogels. However, upon cooling, the physical interaction among the organogelators prevails and the organogels revert to the more stable configuration.^85^ In the work reported by Esposito et al., a 12-hydroxystearic acid-based organogel as an injectable implant showed a sol to gel transition when the temperature increased from 25 to 37 °C due to the formation of a three-dimensional self-assembly of organogelator molecules, which was driven by noncovalent forces such as London dispersion forces and hydrogen bonding (Figure 7c).^86,87^

#### 2.2.3 Microscopic Characterization

A compound optical microscope and an inverted phase-contrast microscope were used to analyze the microstructures of the samples. The reported work on transdermal delivery of a tamoxifen-loaded pluronic lecithin organogel showed the formation of micelles. Optical microscopy revealed the uniform dispersion of micelles within the pluronic lecithin organogel, while the TEM photograph of the pluronic lecithin organogel presented individual micelles.^88^

#### 2.2.4 pH Determination

The pH of the prepared samples for topical formulations should lie in the range of 4.5–6 (skin pH) to prevent irritation to the skin. In one reported work, the pH for the developed ketoconazole-loaded organogel formulations was found in the range between 6 and 6.8, which is acceptable for skin preparations.^89^ The organogel formulations developed for both pluronic F127 and PLO were found to be slightly acidic, which again represents an appropriate pH for topical application.^90^ In the work reported on a lecithin-based organogel using sunflower oil as the polar phase, the pH of the organogel was found to be 6.3.^91^ The pH of an etodolac-loaded organogel was found to be in the range of 5.1–6.2.^92^

#### 2.2.5 Rheological Behavior

Organogels are three-dimensional structures that are formed due to the physical interactions among the gelator molecules. The organogels behave like a solid at lower shear rates and hence show an elastic property.^93^ The rheological behavior of the fabricated organogels is significantly dependent on the interactions between the individual compounds, mainly the polymer and organogelators used. Organogelators have shown synergistic interactions, with higher values of hardness and moduli (elastic modulus *G*′ and loss modulus *G*″).^94^ As the shear stress progressively increases, the intermolecular connections within the fiber structures gradually decrease until the shear stress surpasses a critical threshold, leading to the disruption of intermolecular interactions and subsequent flow of the organogels. This behavior may be best explained by the plastic flow behavior.^79^ Dynamic stress sweep studies performed for a naproxen organogel showed a higher *G*′ value compared to *G*″, which clearly indicated elastic behavior of the fabricated gel.^95^

#### 2.2.6 Swelling

Gels can swell by absorbing liquid with an increase in volume (Figure 8b). Solvent penetrates the gel matrix so that gel–gel interaction is replaced by gel–solvent interaction. The occurrence of limited swelling is typically attributed to the presence of a partially cross-linked gel matrix, which hinders complete disintegration.^85^ The swelling studies performed for a nanocomposite organogel in a mixture of different organic solvents (isopropanol, 35% v/v; isooctane, 45% v/v; and acetone, 20% v/v) showed 87% of organic solvents were retained within the swollen matrix (Figure 7d).^96^ In another study, the swelling studies for fabricated aromatic nonpolar organogels in different hydrocarbon solvents were reported. Effective incorporation of aromatic interactions into the polymer–solvent system resulted in a significant increase in the swelling ratio. This, in turn, facilitates the efficient plasticization of the polymer networks.^97^

#### 2.2.7 Water Content

A study was performed to analyze the lecithin organogel system using near-infrared (NIR) spectroscopy. The focus of the study was to evaluate the water absorption peaks. The results showed that the water has strong absorption peaks at 1918 nm due to H–O–H stretching overtone, which are easily detectable and quantified (Figure 7e).^98^

#### 2.2.8 *In Vitro* Drug Release

The release of drugs from the organogel through various membranes was determined using a Franz diffusion cell.^99^ The work reported on the preparation and characterization of a lecithin and palm oil-based organogel containing metronidazole as a model drug showed controlled release of the drug for 12 h, which was much less as (40% w/w) when compared to the formulations (45%–65% w/w).^100^ In another study, the effect of an organogelator on the release profile of a model drug, ibuprofen, was studied. The results showed that the release rate of ibuprofen from the organogel decreased with an increase in the amount of gelator, 12-hydroxystearic acid (12-HAS). *In vivo* studies showed suppression of rapid absorption for the organogel formulation when compared with an ibuprofen suspension.^101^

#### 2.2.9 Skin Irritation Study

The objective of this study was to assess the ability of a drug-loaded organogel to induce skin irritation in rats in comparison to the skin of healthy individuals (Figure 8c and d). The work evaluated whether a pluronic lecithin organogel containing mefenamic acid showed skin irritation in a study that was performed in each group of six rats. The albino rats were used after obtaining the approval of the Institute Animal Ethics Committee (MMCP/IAEC/12/25), and the experiments were performed in accordance with all regulations. The hair on the dorsal region of the rat was removed using a depilatory cream. An area of 4 cm^2^ was marked on the dorsal surface of the skin. After 24 h of hair removal, the formulations were applied on the skin’s surface at a dose of 100 mg per rat once a day for a duration of 7 days. The area was occluded. The absence of skin irritation in a gel composition is deemed to be acceptable. To this end, no signs of erythema, edema, or skin reddening were seen. All gel formulations examined were observed to be devoid of any indications of discomfort.^102^ In another work, the skin irritation studies were performed for the pluronic lecithin organogel containing amphotericin B. The fur was removed from the dorsal surface of the mice in a similar fashion as discussed previously. After 24 h, the gel formulation was applied at a dose of 100 mg per rat. Notably, the calculated primary irritation index was found to be 0.027, without any signs of erythema at the end of 24 h.^103^

#### 2.2.10 Phase Behavior of a Three-Component System

A phase diagram was constructed for the lecithin-oil–water system (Figure 7f). The phase diagram showed the distinct phases as a function of composition factors and temperatures. In an organogel system, lecithin, oil, and water concentrations are crucial. Initially, lecithin micelles were generated by utilizing water-in-oil micron emulsions with low water concentrations. This resulted in optical clarity and low viscosity. As the quantity of water increased, the microemulsion underwent a transformation into a viscous gel. Consequently, the organogel is sometimes referred to as a microemulsion-based organogel. The other properties of the organogel system, including cloudiness, isotropy, optical transparency, and viscosity, can also be evaluated from the phase diagram. It was found that too much water makes the system murky; therefore, water concentration is particularly important in the development of a clear organogel.^104,105^

### 2.3 Factors Influencing the Organogel Formulation

To create the ideal conditions for the successful development of an organogel formulation, it is important to keep an eye on and understand variety of variables that affect the formation of organogels. These parameters and their known effects on the resulting organogel network are listed in Table 2.

**Table 2:** Various Factors Influencing Organogel Formulation

sl no.	parameters	factors	influence
1	solvent	water presence	stability and confirmation^83^
		cosolvent presence	morphology and confirmation^108^
		nature of solvent	morphology, confirmation, and optical properties^109^
2	organogelators	molecular weight, concentration, and charge	confirmation and mechanical and rheological properties^110−112^
3	adjuvants	salt and surfactant addition	morphology and confirmation^113−115^

## 3 Types of Organogels

Organogels are typically categorized based on the type of the organogelator. Various type of organogels with their applications can be classified based on the nature of intermolecular interactions (chemical or physical), solvent type, and synthesis techniques. To this end, various types of organogels are broadly discussed below.

### 3.1 Supramolecular Organogel

These organogels are created from low molecular mass gelators. This class of organogels has provided a field of interest for the creation of various gels with technological applications, including being sensitive to external stimuli like light. Supramolecular organogel systems with regulated self-assembled structures exhibit remarkable thermoreversibility and mechanical properties. Controlled drug delivery may be possible with these organogels. They provide a variety of functions as carriers.^116^ Preparation of a three-dimensional supramolecular organogel was reported. The gel was efficiently formed by mixing a 1,1,1,3,3,3-hexafluoro-2-propanol (HFIP) solution of cyclodextrin (CD) with 1- or 2-butanol via the formation of three-dimensional hexagonal nanostructures composed of head to-tail CD channel assemblies.^117^ These nanometer-sized hexagonal prisms were believed as key intermediates for the formation of supramolecular organogel.

### 3.2 Limonene GP1/PG Organogel

**Figure 9:**
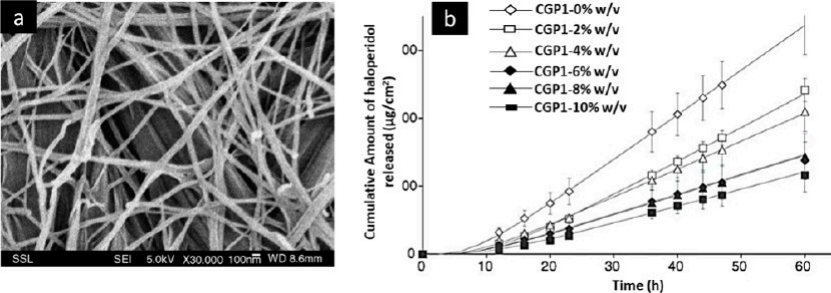
(a) Formation of fibrous network and (b) *ex vivo* skin permeation profile of a limonene GP1/PG organogel formulation containing haloperidol. Adapted and revised with permission from ref (119). Copyright 2006 Elsevier.

Limonene, a terpenoid with exceptional penetrating strength, is employed in transdermal drug administration systems to increase the bioavailability of medications.^118^ This organogel is made by combining limonene, propylene glycol (PG), and an appropriate quantity of dibutyllauroylbutamide (GP1), an amino acid type of organogelator. The mixture is then incubated at 120 °C. It transforms into a white gel after cooling. The GP1/PG organogels tends to enhance gel moduli due to the inclusion of limonene, which indicates the increased physical stability of the gel.^119^ Transdermal delivery of haloperidol was reported using a limonene-based organogel containing two types of major organogelators, namely, dibutyllauroylglutamide and propylene glycol, at varying concentrations. The SEM and *ex vivo* studies showed the formation of a fibrous network and increased permeation across the human abdominal skin (Figure 9a and b).

### 3.3 Sorbitan Monostearate Organogel

**Figure 10:**
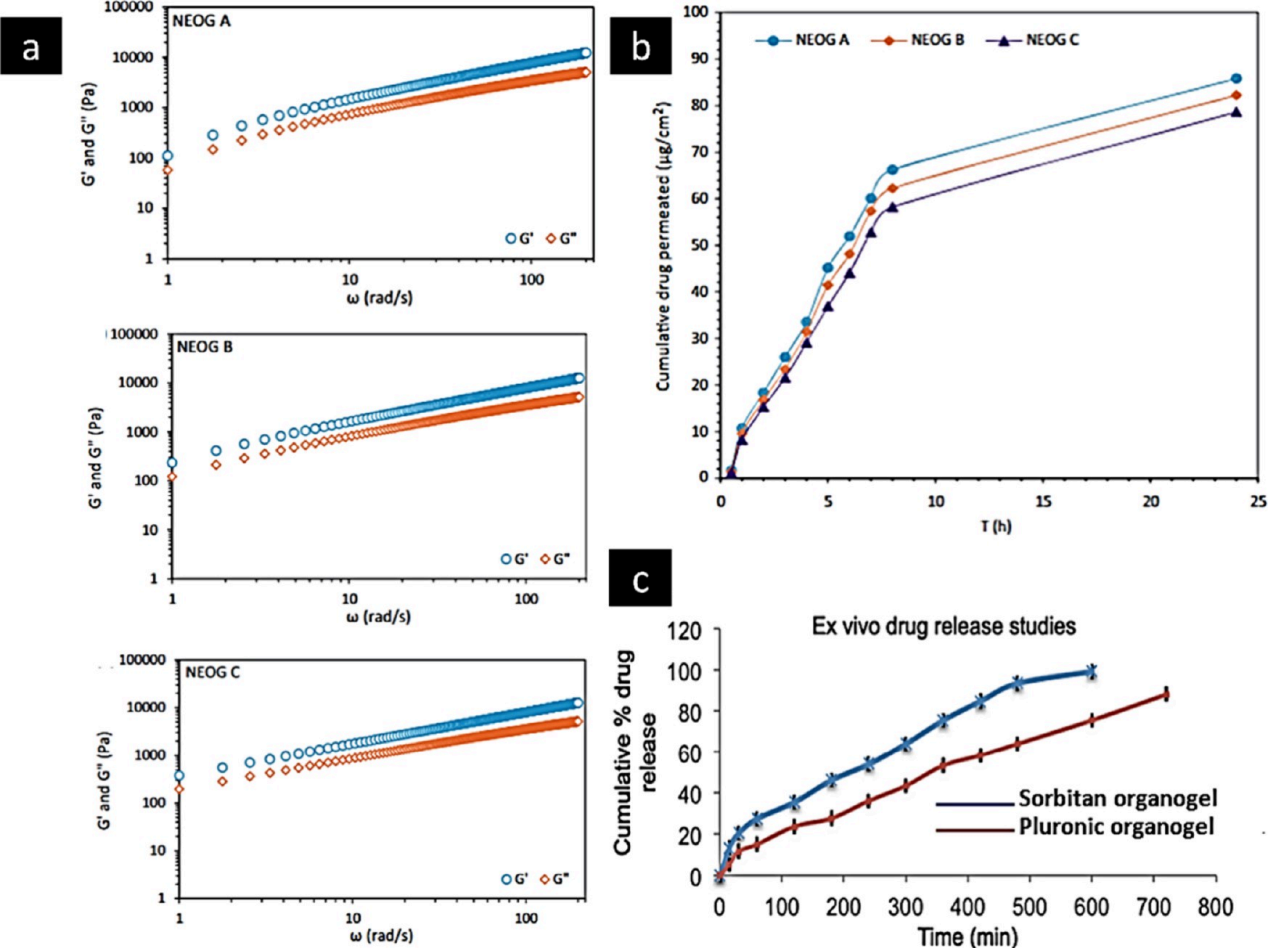
(a) Rheological behavior and (b) skin permeation profile of an acyclovir sorbitan-based organogel formulation. Adapted and revised with permission from ref (121). Copyright 2016 Taylor & Francis. (C) Profile of an amphotericin sorbitan-based organogel formulation. Adapted and revised with permission from ref (103). Copyright 2020 SciELO Brazil.

Sorbitan monostearate organogels formed by mixing of sorbitan monostearate (Span 60) and sorbitan monopalmitate (Span 40) in an apolar solvent at low concentrations. As compared to Span 40, the Span 60 gels were more stable. These gels are prepared by heating the gelators and liquid medium, then cooling the mixture to form a suspension, which results in an opaque white semisolid gel.^120^ A nanoemulsion-based organogel containing sorbitan ester was fabricated for the transdermal delivery of acyclovir.^121^ The developed organogel showed good storage and loss moduli with a compact and dense network (Figure 10a), which was hypothesized for sustained and site-specific delivery of drugs. The *ex vivo* skin permeation data across rat skin showed the sustained release of acyclovir with greater retention within the skin (Figure 10b). In another study, an amphotericin-loaded sorbitan monostearate organogel was developed for mucocutaneous fungal infections.^103^ A comparative study was performed with pluronic-based organogel formulation (PLO). *Ex vivo* skin permeation studies across porcine skin showed cumulative drug release of 99.3% when compared with PLO gel (Figure 10c).

### 3.4 Eudragit Organogel

**Figure 11:**
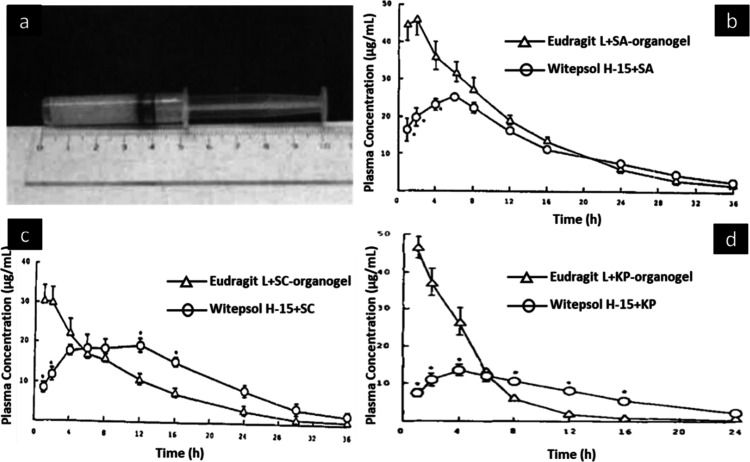
(a) Designed syringe filled with organogel for rectal administration. Photograph courtesy of Goto. Copyright 2024. *In vivo* pharmacokinetic profile of 30% w/w Eudragit organogel formulation and Witepsol suppositories for (b) salicylic acid (SA), (c) sodium salicylate (SC), and (d) ketoprofen (KP). Adapted and revised with permission from ref (47). Copyright 1991 Elsevier.

Eudragit organogels differ from other organogels in that they are composed of a mixture of Eudragit (L or S) and polyhydric alcohols such as glycerol, propylene glycol, and liquid polyethylene glycol, with high Eudragit concentrations (30 or 40% w/w). To make drug-containing gels, drugs including salicylic acid, sodium salicylate, procaine, or ketoprofen were dissolved in propylene glycol, and the solution was poured into Eudragit powder. This was mixed with a pestle for 1 min (Figure 11a). The viscosity of the gel increased with increasing Eudragit concentrations and decreased with increasing drug content.^47^*In vivo* evaluation studies performed in the rabbits showed sustained plasma concentrations for salicylic acid, sodium salicylate, and ketoprofen drugs for 30% w/w organogel formulations (Figure 11b–d).

### 3.5 Microemulsion–Gelatin-Based Organogel

**Figure 12:**
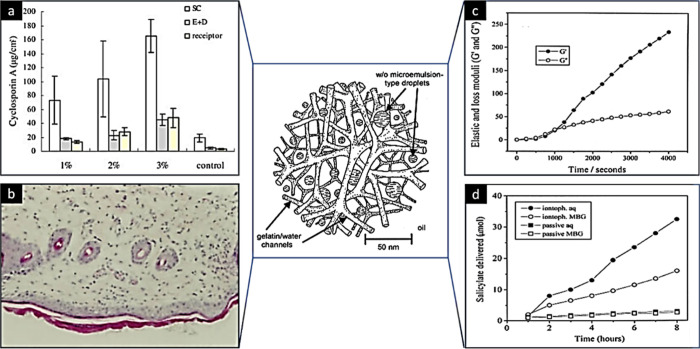
(a) *Ex vivo* skin retention and permeation of cyclosporine A (CsA) in different concentrations and (b) H&E-stained images captured for a CsA-loaded organogel (1% w/w). Adapted and revised with permission from ref (122). Copyright 2007 Elsevier. (c) Rheological behavior and (d) *ex vivo* skin permeation profile of a MBG organogel loaded with sodium salicylate. SC, stratum cornuem; E, epidermis; and D, dermis. Adapted and revised with permission from ref (123). Copyright 1999 Elsevier.

In microemulsion-based gels (MBGs), gelatin is a hydrophilic polymer that gels water. To manufacture MBGs, solid gelatin was dissolved in a hot W/O microemulsion and subsequently chilled. In MBGs, gelatin dissolves in the water droplets of the W/O microemulsion, and freezing the solution causes the water droplets to gel, resulting in clouding and perhaps phase separation. A microemulsion–gelatin-based organogel was reported that showed greater retention and moderate percutaneous penetration of cyclosporine A in both *ex vivo* and *in vivo* studies performed across the skin of SD rats (Figure 12a).^122^ The drug-loaded organogel did not show any gross changes to the skin, which was evident from hematoxylin and eosin (H&E) staining, and therefore the application of organogel was considered to be relatively safe (Figure 12b). In another study, MBGs containing surfactants including Tween 85 and isopropyl myristate showed a gradual increase in the elastic modulus (*G*′) over the loss modulus (*G*″) with an increase in the concentration of solid gelatin (Figure 12c). Also, enhanced delivery of sodium salicylate was achieved using the iontophoresis technique across porcine skin (Figure 12d).^123^

### 3.6 Pluronic Lecithin Organogels (PLOs)

**Figure 13:**
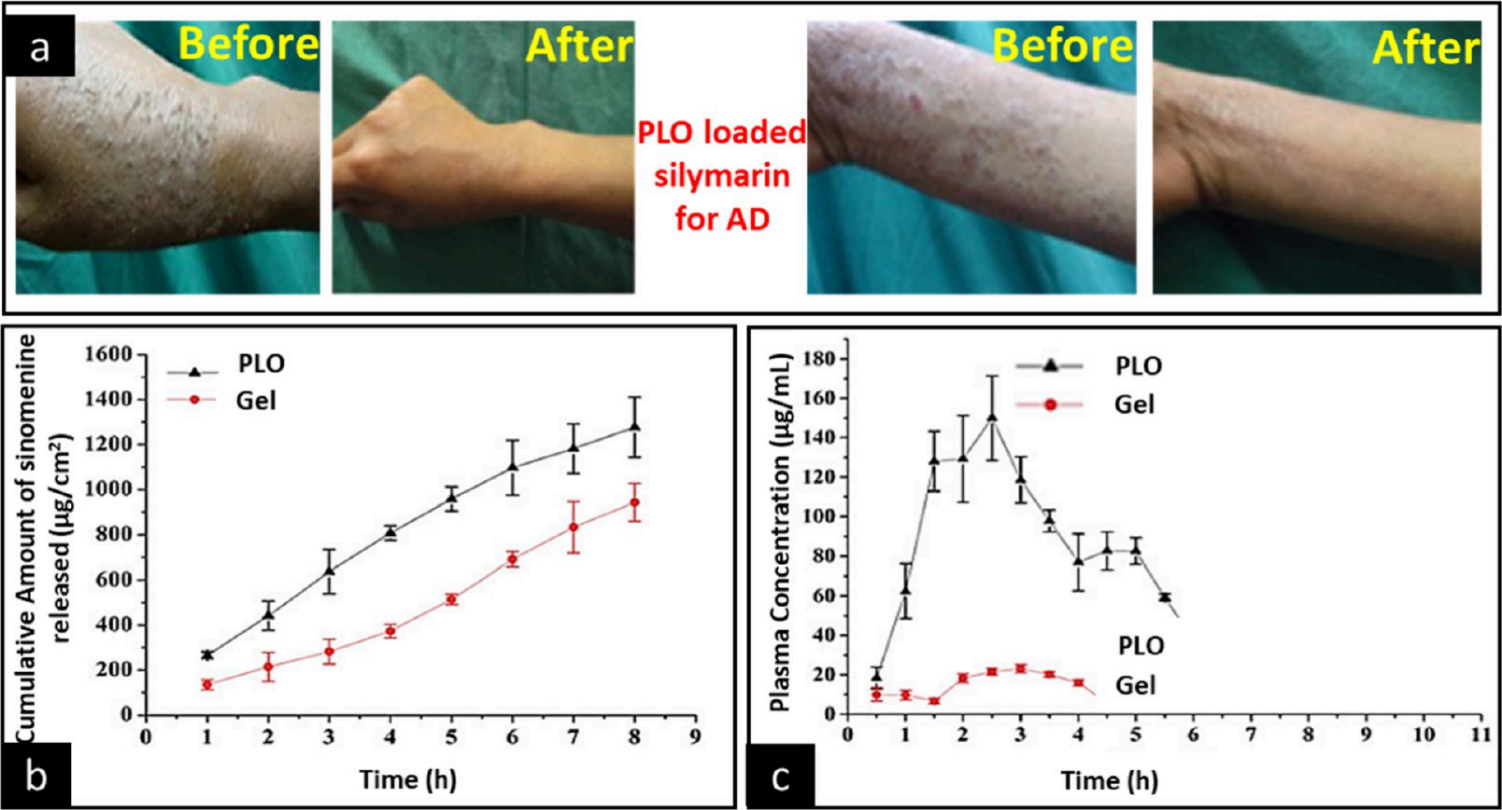
(a) Application of a silymarin pluronic lecithin organogel (PLO) on the human volunteers for the treatment of atopic dermatitis (AD). Adapted and revised with permission from ref (125). Copyright 2016. (b) *Ex vivo* skin permeation and (c) *in vivo* studies of a sinomenine-based PLO across abdominal rat skin. PLO, pluronic lecithin organogel. Adapted and revised with permission from ref (126). Copyright 2015 Taylor & Francis.

Pluronic lecithin organogels (PLOs) are translucent yellow gels mainly composed of isopropyl palmitate, soy lecithin, water, and the hydrophilic polymer. Pluronic F127 (a hydrophilic polymer that gels water) and the larger volume of water compared to the oil distinguish PLO from its progenitor, lecithin gels. PLO is not an organogel in the traditional sense, although it is referred to as such because of its name. To assist in stabilizing the initial lecithin organogel formulation, pluronic F127 was added. PLOs are used as a topical or transdermal drug carrier for a variety of drugs, including haloperidol, prochlorperazine, secretin, and some hormones. PLOs have also been investigated/proposed as an oral cavity and mucosa delivery mechanism.^124^ In another reported work, a PLO that loaded silymarin with lecithin showed effectiveness for the treatment of atopic dermatitis (Figure 13a).^125^ The sinomenine loaded in the PLO organogel was effectively delivered across the porcine skin. *In vivo* studies showed greater retention when compared with the marketed gel formulation (Figure 13b). Pharmacokinetic studies revealed significantly higher *C*_max_ in blood plasma, and the concentration–infinity curve values of the PLO gel were also 3.29-fold higher than those of the marketed gel formulation (Figure 13c).^126^

### 3.7 Lecithin Organogel (LO)

Lecithin is a mixture of phosphatidylcholines with acyl chains of different lengths and degrees of unsaturation. It is also a mixture of triglycerides and other nonphospholipid compounds.^127^ Lecithin or phosphatidylcholine is the most predominant phospholipid in biological systems and is generally refined from soybeans and egg yolk. Lecithin organogels (LOs) are considered favorable drug delivery vehicles due to their biocompatibility and amphiphilic nature, which aids in the solubility of several drug classes and therefore enhances penetration.^128^ LOs are isotropic gels that contain phospholipids, an apolar solvent, and a polar solvent that is thermodynamically stable, transparent, viscoelastic, and biocompatible. LOs are jelly-like phases consisting of a three-dimensional network of reverse cylindrical (polymer-like) micelles that immobilize the external organic phase, changing it from a liquid to a viscous gel.^129^ In the first description of LOs reported, solutions of lecithin formed transparent gel-like lipid aggregates in organic solvents including cyclic alkanes, fatty acid esters, and amines, among others.^130^ The authors evaluated different conditions that might allow soy lecithin to produce reverse micelles. In these studies, water was added to various organic solutions of pure soybean lecithin. The introduction of minute amounts of water into nonaqueous solutions of soy lecithin resulted in a rapid increase in viscosity, with values from 104× to 106× higher than that of the original nonviscous solution. This led to a transformation of the solution into a gel or jelly-like state. LOs may efficiently moisturize the skin even in a lipid-rich environment because of their unique architecture. Topical administration has the major advantage of bypassing first-pass metabolism. Another benefit of topical preparations is that they avoid the risks and inconveniences of intravenous therapy, as well as the many circumstances of absorption, such as pH changes, enzyme presence, and stomach emptying time, reducing medication dosage frequency.^131^

#### 3.7.1 Composition of LOs

**Figure 14:**
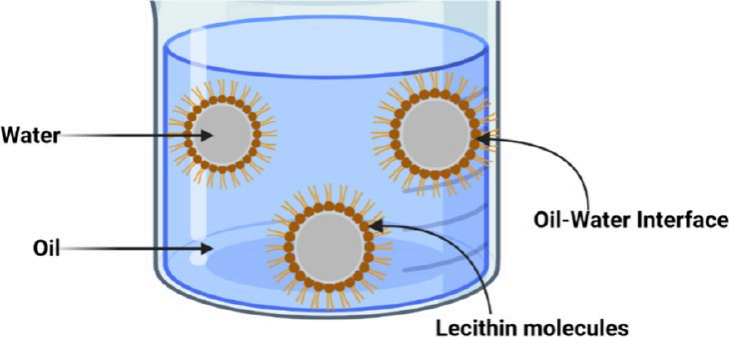
Schematic illustration representing the organization of lecithin molecules inside micellar structures.

LOs are made up of a polar agent (typically water) and a biosurfactant (lecithin) that functions as a gelling agent, as well as a nonpolar organic medium as the external or continuous phase, as shown in Figure 14. Lecithin comprises phospholipid molecules that self-assemble to form the organogel’s microstructure. The critical packing parameter can be impacted by the unsaturated nonpolar segment of lecithin molecules. It assists in the formation of reverse micellar structures and turns micelles into three-dimensional long tubular networks.^132^ An organogel relies on an organic solvent that provides the appropriate solvent action for the drugs and lecithin and thus supports its skin-penetration-increasing feature.^27^ Ethyl laureate, ethyl myristate, isopropyl myristate, isopropyl palmitate, cyclopentane, cyclooctane, *trans*-decalin, *trans*-pinane, *n*-pentane, *n*-hexane, *n*-hexadecane, and tripropylamine are just several of the organic solvents used for LOs. Organic solvents that are biocompatible and biodegradable are quite often favored due to their safety during use. As solvents in LOs, natural oils like soybean oil, sunflower oil, rapeseed oil, and mustard oil have desirable properties.^133^

#### 3.7.2 Gelation Mechanism of LOs

**Figure 15:**
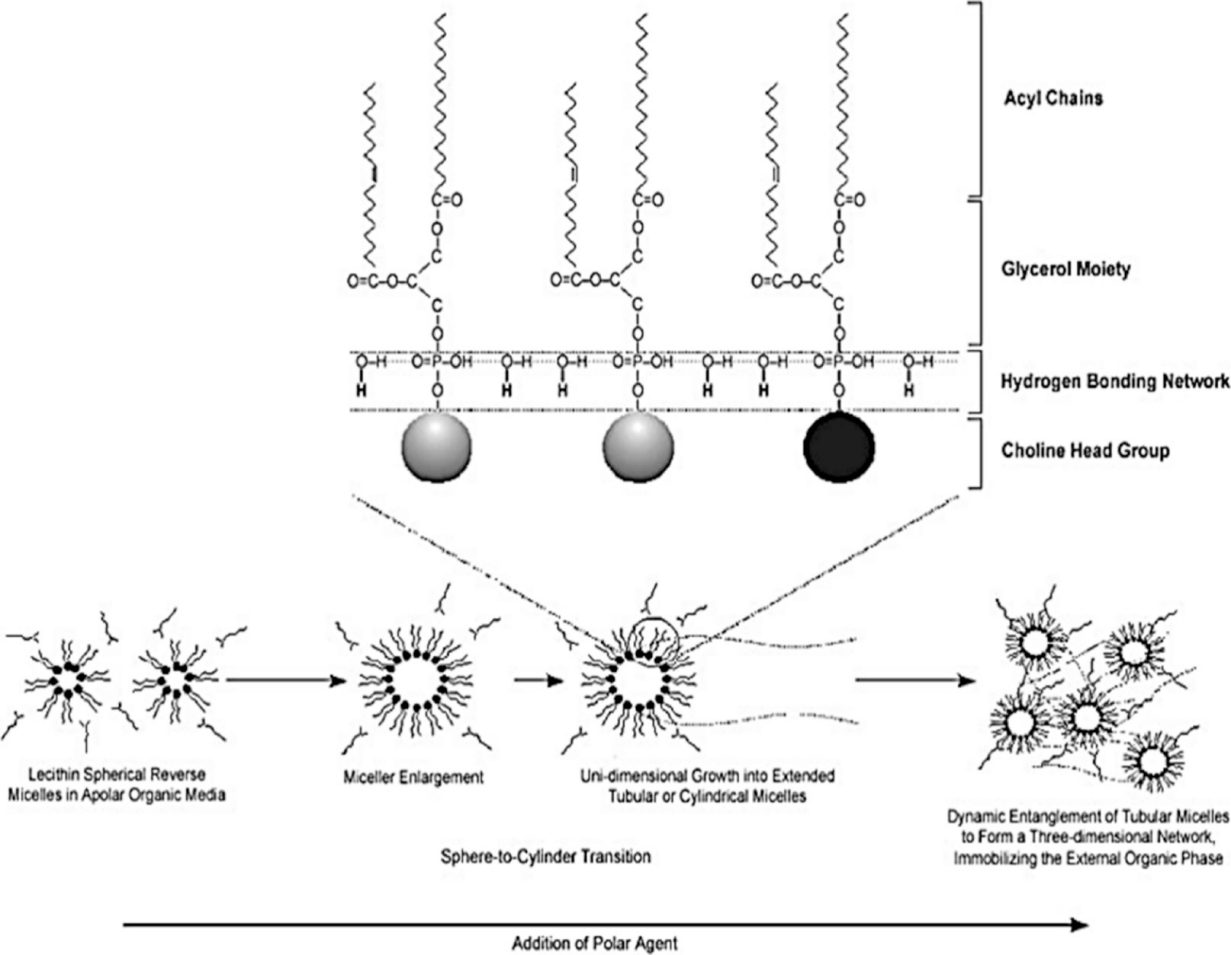
Formation of a three-dimensional network of reverse cylindrical micelles in a lecithin organogel is facilitated by the establishment of hydrogen bonding interactions between lecithin molecules and polar solvent molecules. Adapted with permission from refs (72) and (138). Copyright 2005 American Association of Pharmaceutical Scientists and 1995 Elsevier, respectively.

The process of organogelling, or the gelation of lecithin solutions in organic solvents, is initiated through the inclusion of a polar solvent. When lecithin is dissolved solely in nonpolar fluids, it undergoes self-assembly to form reverse spherical micelles.^134^ The significant uniaxial expansion of these spherical reverse micelles and subsequent conversion into tubular or cylindrical micellar aggregates (known as sphere-to-cylinder transformation) is initiated by the introduction of minute and crucial quantities of a polar additive, as depicted in Figure 15. Upon addition, the molecules of a polar solvent form stoichiometric bonds with the hydrophilic head region of the lecithin molecules. This binding arrangement results in the bridging of two neighboring lecithin molecules by a single polar molecule.^135^ This phenomenon results in the creation of linear networks, which are generated by the hydrogen bonds established between polar molecules and phosphate groups of lecithin molecules. Consequently, this leads to the unidirectional growth of lecithin reverse micelles in a one-dimensional manner. An additional increase in the quantity of polar additive leads to the generation of pliable, elongated tubular micelles with radii ranging from 2.0 to 2.5 nm and a length spanning from hundreds to thousands of nanometers.^136^ Once the expanded micelles reach a sufficient length, they undergo a process of overlapping, entanglement, and the formation of a temporary three-dimensional network.^133,137^ This transition represents a shift toward a system that exhibits higher viscosity and viscoelastic properties. Instead of a low-viscosity solution, a gel-like phase is formed. The LO phase contains a significant proportion (approximately 85% by weight) of an external phase, which is an organic liquid trapped within the interstitial spaces between the interconnected reverse micelles. The formation of a hydrogen bonding network facilitated by polar additives and phosphate groups is also accompanied by increased rigidity of the phospholipid molecule in the vicinity of the phosphate group and glycerol residue. This enhanced stability contributes to the formation of the micellar aggregates.

#### 3.7.3 Application of LOs in Topical and Transdermal Delivery of Therapeutic Molecules

The scope of organogels has expanded more in the administration of drugs either topically or transdermally. Delivery of drugs in the skin layer (cutaneous or dermal delivery) is beneficial as it is noninvasive, easy to administer, and also avoids the first-pass metabolism of the active ingredient(s).^139^ Lecithin organogels are particularly fascinating systems because of their biocompatibility and amphiphilic character.^140^ To this end, lecithin organogels with great potential for transdermal application of Etodolac (ETD) was developed.^92^ The result showed enhanced permeation with sustained release for up to 6 h. Skin irritation and histological studies showed that the fabricated gel was nonirritating and nontoxic. Synthesized castor oil organogel nanoparticles containing ketoconazole or indomethacin with distinctive ionization characteristics were reported.^141^ The entrapment efficiency of both compounds was found to be excellent, and stability experiments revealed that there was minimal drug leakage observed during storage. Moreover, the *in vitro* dialysis findings demonstrated the prompt release of the drug from the organogel nanoparticles. In relation to biocompatibility, stability, and scalability, these systems present themselves as potentially feasible substitutes for nanoemulsions or solid lipid nanoparticles (SLNs) in the context of lipophilic drug delivery.

The researchers reported the fabrication of conventional organogels and microemulsion-laden organogels, also known as microemulsion organogels, for the purpose of topical delivery of the lipophilic drug lidocaine.^142^ The conventional and microemulsion organogels containing lidocaine exhibited viscoelastic properties characterized by a higher degree of elasticity. The conventional organogel containing lidocaine demonstrated the highest viscoelasticity and the slowest release rate. In contrast, the microemulsion organogel consisting of Tween 20/ethanol (4:1 v/v) exhibited lower viscoelasticity and a higher rate of drug release. Taken together, it was concluded that the drug penetration rate was improved by utilizing a microemulsion-laden organogel as a carrier system due to the factors including small droplet size and large drug payload. These are attributed to the high solubility of lidocaine in microemulsions. Consequently, this phenomenon also showed a depot effect, facilitating the accumulation of lidocaine inside the layers of the skin for an extended duration.

In another study, the effect of a hyaluronan (HA) microparticle-loaded lecithin organogel containing caffeine on cellulite (dermal disorder) was reported.^143^ The microparticle mixtures that were fabricated (size 33.97 ± 0.3 μm, span <2; encapsulation efficiency 88.56 ± 0.42%) showed optimum viscosity after they were homogeneously distributed in lecithin organogels. *Ex vivo* studies showed that the concentration of caffeine in the microparticle-loaded organogels was found to be twice as high as that in the aqueous solution after 24 h. The observed phenomenon pertained to the continuous release of caffeine from the microparticles. Therefore, it was concluded that the utilization of a lecithin organogel incorporating HA-encapsulated microparticles can be a viable option for the development of an effective topical drug delivery systems for caffeine. Furthermore, the potential synergistic effect resulting from the combination of these moieties in combination with a carrier system presents a viable strategy for the development of long-acting treatments for cellulite.

One of the limitations encountered with the topical semisolid dosage form is its tendency to be easily removed shortly after application, leading to reduced effectiveness of the active ingredient. To this end, a study involving the preparation of topical organogels using various oils was devised and reported the controlled release of miconazole nitrate.^144^ Controlled release of organogels was achieved when they were formulated using glyceryl monostearate at a concentration of 15% w/v. Experiments using a Franz diffusion cell revealed that 85% of the drug was retained on the skin, suggesting its potential for topical antifungal therapy. After a duration of 24 h, it was observed that only 15% of the drug had been released. Consequently, a significant proportion of the drug was retained within the skin, indicated its availability for therapeutic activity.

**Figure 16:**
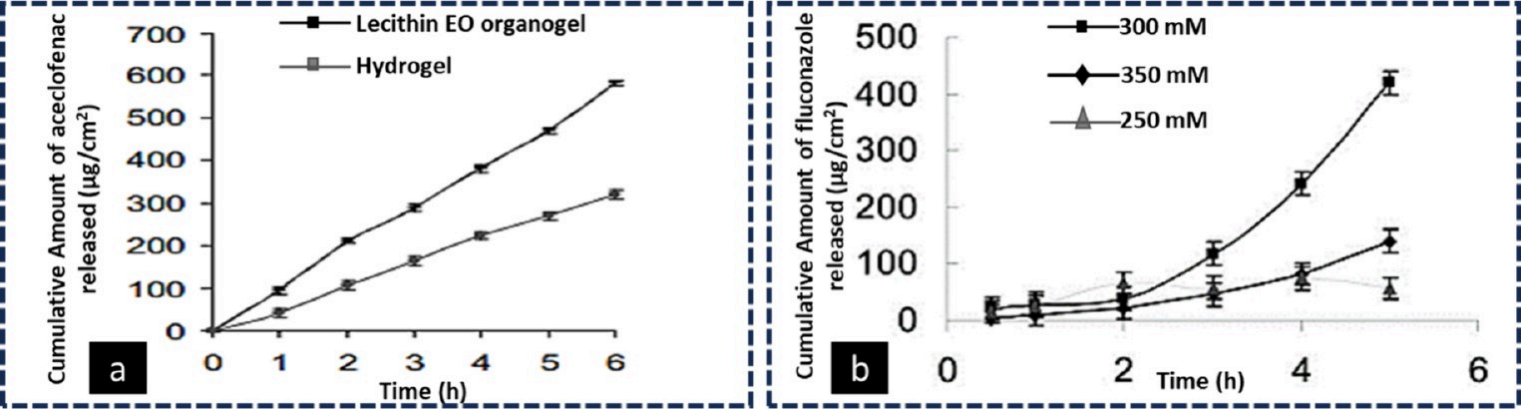
*Ex vivo* skin permeation profiles of (a) aceclofenac and (b) fluconazole from lecithin organogel formulations. Panel (a) adapted and revised with permission from ref (145). Copyright 2009 Bentham Science. Panel (b) adapted and revised with permission from ref (146), Copyright 2009 Bentham Science.

Several bioactive compounds used in topical skin therapy can be delivered using lecithin organogels. An aceclofenac-loaded lecithin organogel was prepared containing ethyl oleate (EO).^145^ The fabricated organogel was compared with the conventionally prepared Carbopol gel. The *ex vivo* studies performed across the abdominal skin of rats showed enhanced permeation of aceclofenac from the lecithin organogel due to the presence of lecithin, which affected the lipids in the SC by altering their arrangement and disordering them transiently (Figure 16a). In another study, a lecithin organogel containing fluconazole was also prepared, and its antifungal activity was reported.^146^ The lecithin was used in different concentrations (250, 300, and 350 mM). Figure 16b shows the *ex vivo* skin permeation of fluconazole across the rat skin, which was enhanced from the lecithin organogel with a concentration of 300 mM due to the increased thermodynamic activity of the drug with increased lecithin concentration until it reached the limiting value. In contrast, the permeation was decreased from the lecithin organogel with a concentration of of 350 mM due to the formation of fiber-like entangled micelles with higher viscosity. The optimized lecithin organogel showed increased antifungal activity due to the surfactant action of lecithin.

## 4 Method of Formation of LOs

### 4.1 Fluid-Filled Fiber Mechanism

In the fluid-filled fiber mechanism, the organogel was made by compounding the surfactant combination with an apolar solvent. In the first stage, reverse micelles will develop, and the addition of water to these micelles will result in tubular reverse micelles. With the addition of more water, a three-dimensional network structure forms, immobilizing the apolar solvent.^147^

### 4.2 Solid Fiber Mechanism

In the solid fiber mechanism, the synthesis of an organogel begins with the heating of an apolar solvent and a solid organogelator. Allow for ambient temperature to reach the nonpolar solution. When organogelators are cooled, they precipitate as fibers, generating a three-dimensional networked structure that immobilizes the polar solvent through physical interactions.^44^

### 4.3 Hydration Method

Hydration is one of the simplest techniques to prepare an organogel. In this case, adding water directly to the inorganic chemical may result in the formation of organogel. In addition to the water carrier, agents such as propylene glycol, propyl gallate, and propyl hydroxyl cellulose are added to improve gel formation.

### 4.4 Homogenization and Microirradiation

Organogels can be produced alternatively by mixing the polymer–solvent dispersion for 5 min at 24 000 rpm. This homogenized mixture can be used to make gels in two ways:

(a) Heating the homogenated dispersion in a water bath at 80 °C with 200 rpm mechanical stirring is the first approach. After 10 min of stirring, it will form a uniform translucent gel system.
(b) The second method is microirradiation. The homogenized solution was put in a Petri plate and microirradiated for 2 min, yielding a transparent gel structure.^148^ Resveratrol organogels containing Carbopol 940 in various kinds of polyethylene glycol (PEG) were prepared using high-speed homogenization. Subsequently, the samples were subjected to microirradiation. The rheological properties of the obtained gel formulations were examined, and it was observed that they exhibited the expected behavior characteristic of a non-Newtonian fluid.^149^ In another study, a triclosan organogel was prepared using Carbopol 974 NF in PEG 400. Carbopol was homogeneously dispersed in PEG 400 at different concentrations ranging from 2% to 4%. The dispersion that was obtained was homogenized at a speed of 24 000 rpm. After heating and continuous agitation, in the second approach, the dispersion was again subjected to microirradiation at a power of 1200 W for a duration of 1 h. The findings of the study indicated that microwave heating proved to be an appropriate method for the preparation of Carbopol organogels.^148^

## 5 LOs: A Sword to Mitigate Skin Diseases

**Figure 17:**
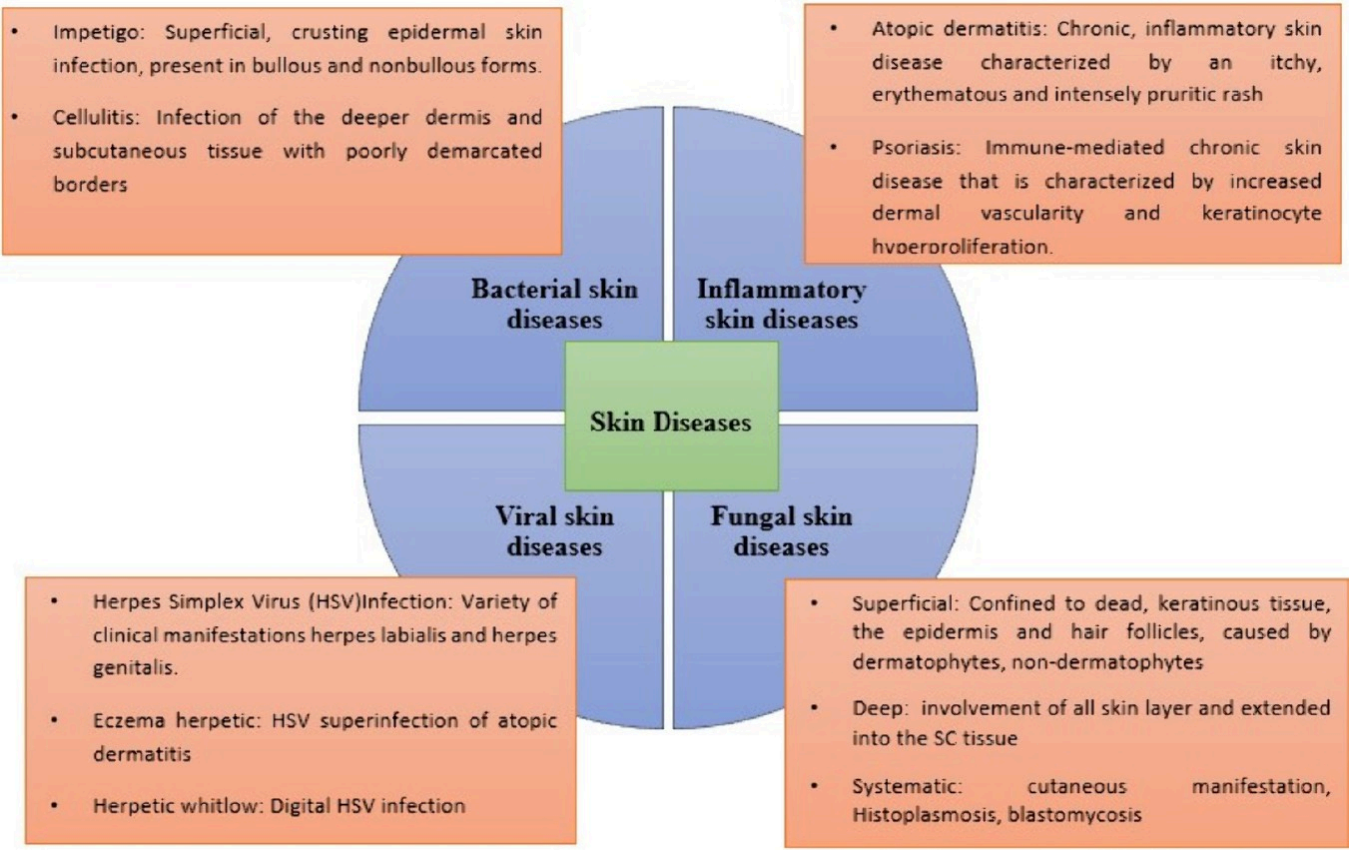
Schematic illustration of the application of lecithin organogels in the management of severe skin diseases.

Despite the skin’s many important functions (insulation, temperature regulation, element and molecule absorption, sensation, storage, and vitamin D synthesis), it can be affected by a variety of conditions, including rashes, viral, bacterial, fungal, and parasitic infections; pigmentation disorders; trauma (i.e., an injury to the skin caused by a blow, cut, or burn); tumors; and cancer (Figure 17).^150^ Many skin-related issues, particularly those connected to infectious skin disease, are difficult to cure. These issues are, in fact, dependent on the pathogens implicated, the integrity of the skin layers and their structures, and the patient’s underlying medical state.^151^ The application of a lecithin organogel containing a therapeutic moiety has been explored in the field of various skin diseases (Table 3). Chronic inflammatory skin illnesses, including psoriasis, atopic dermatitis, and allergic contact dermatitis, are caused by inflammatory T cells infiltrating the lesions and producing more cytokines.^152^ The preparation and *in vivo* studies reported for a lecithin organogel loaded with α-mangostin with antimicrobial activity showed significant reduction in the bacterial number caused by *Staphylococcus pseudintermidus*. The study also showed partial restoration of the epidermal barrier and suppressed the expression of cytokine genes associated with pro-inflammatory Th1, Th2, and Th17 in skin lesions induced by *S. pseudintermedius* in a murine model.^153^ In another work, a lecithin organogel loaded with halobetasol propionate showed enhanced and deep layer permeation across rodent skin, which was confirmed by confocal microscopy using a fluorescent marker. To this end, it was hypothesized that this formulation would not aggravate bacterial skin diseases like atopic dermatitis, which becomes more severe and relapses with time, and could translate to better management.^154^

**Table 3:** Examples and Applications of Various Types of Drug-Loaded Organogels in Skin Diseases^a^

sl no.	drug	type of organogel	disease
1	ketoconazole	PLO	antifungal^89^
2	resveratrol	PLO	wound healing^149^
3	fluocinolone acetonide	PLO	psoriasis^155^
4	propolis	PLO	wound healing^156^
5	mefenamic acid	PLO	NSAIDs/anti-inflammation^102^
6	sinomenine	PLO	arthritis^126^
7	acyclovir	PLO	antivirus^157^
8	diltiazem HCl	PLO	antihypertensive^158^
9	halobetasol propionate	PLO	atopic dermatitis^154^
10	ondansetron	PLO	antiemetic^159^
11	silymarin	PLO	atopic dermatitis^125^
12	curcumin	LO	cutaneous pathologies^160^
13	tamoxifen	LO	psoriasis and excessive dermal scarring^88^
14	fluconazole	LO	antifungal^146^
15	nicardipine hydrochloride	LO	antihypertensive^161^
16	etodolac	LO	rheumatoid arthritis^92^

a PLO, pluronic lecithin organogel; LO, lecithin organogel.

*In vitro* transcutaneous delivery of the anti-inflammatory drug ketoprofen in combination with other unsaturated fatty acids from a lecithin organogel containing fish oil was reported.^162^ Studies showed that a substantial amount of ketoprofen was delivered across the full-thickness porcine skin from the lecithin organogel formulation for up to 24 h. In another reported work, the delivery of a topical corticosteroid, fluocinolone acetonide, was sustained from the lecithin organogel formulation as the regimen for skin inflammation.^155^ The delivery of fluconazole and diclofenac diethylamine was enhanced in combination with cellulose derivatives (hydroxypropyl methyl cellulose and hydroxypropyl cellulose) from a lecithin microemulsion-based organogel formulation.^163^ The application of lecithin organogels has also been explored in the field of viral skin diseases. The delivery of mangiferin for the treatment of herpes simplex virus type 1 (HSV-1) was achieved from a lecithin organogel formulation. This gel formulation showed safety after cutaneous administration in human volunteers. Also, *in vivo* efficacy studies showed reduction in the plaque against the HSV-1 KOS strain.^164^

For the first time, skin permeation of terconazole (the most active triazole), was investigated for the dermal application in skin candidiasis. The skin permeation studies showed enhanced transport of the drug across the rat abdominal skin. This drug was loaded in the lecithin-integrated liquid crystalline nano-organogels. Acute irritation studies were performed that showed the absence of any skin inflammation.^165^ In another work, the penetration and retention of a lecithin organogel formulation containing ketoconazole in combination with tea tree oil through the artificial skin membrane was enhanced. Also, the prepared formulation showed enhanced antifungal activity against *Candida parapsilosis*, which proved the designed formulation is a valuable alternative for the treatment of skin fungal diseases.^166^

### 5.1 Atopic Dermatitis

**Figure 18:**
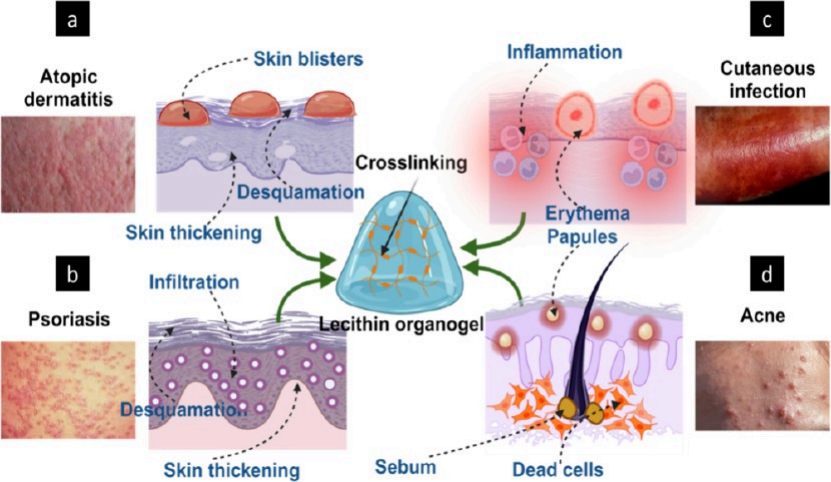
Schematics representing the application of lecithin organogels for dermal or systemic delivery of therapeutics for the treatment of skin disorders: (a) atopic dermatitis, (b) psoriasis, (c) cutaneous infection, and (d) acne.

Atopic dermatitis (AD) is a recurring inflammatory disease characterized by severe pruritic skin that affects many children.^167^ This is linked to an increase in IgE production and a change in pharmacological reactivity. Skin dryness, erythema, and oozing are common signs of AD, and untreated individuals may have crusting and lichenification of the skin. Atopic dermatitis is characterized by an overactive immune response to environmental factors, resulting in dry, itchy skin (Figure 18a). Skin lesions can cause a lot of emotional distress and impair a patient’s quality of life. The severe scratching caused by the disease irritates the skin and disrupts sleep, and the stigma associated with having a visible skin problem also has a negative impact on patients. Stress, allergen contact, scrabbling, and various other factors can cause skin sores.^168^

#### 5.1.1 Pharmacotherapy

AD treatment aims to keep flares under control, shorten their length, and prevent a recurrence. Emollients, topical corticosteroids, and topical calcineurin inhibitors are the most commonly prescribed drugs. Because of their multifaceted anti-inflammatory actions, topical corticosteroids are beneficial in treating AD.^169^ However, the duration and frequency of use are constrained by the local and systemic adverse effects of topical corticosteroids. Due to insufficient penetration into the SC, the effectiveness of existing topical cream-based therapies is restricted.^170^ A silymarin-loaded PLO gel was successfully synthesized and developed. Significant improvement was observed in the patients with AD due to the high penetration capacity and hydration impact of the fabricated organogel.^125^ In another work, a halobetasol propionate (HP) PLO was formulated that showed enhanced skin permeation and skin retention. The proposed formulation is an effective substitute for conventional halobetasol propionate cream due to its enhanced dermal localization of HP. This opens up the potential of lowering the drug’s dosage. Furthermore, the new formulation was nonirritating and had an excellent biocompatibility, a common shortcoming of commercial treatments based on synthetic surfactants.^154^

### 5.2 Psoriasis

Psoriasis is a proliferative and inflammatory illness that affects roughly 2% of the population and is less frequent than AD. Psoriasis is characterized by rapid keratinocyte proliferation, which produces elevated scaly plaques in sites of damage, such as the knees, elbows, buttocks, and knuckles (Figure 18b). The interactions of tumor necrosis factors (TNF-α), interferons (INF-γ), and interleukins (IL-37) have been shown to cause inflammation and epidermal hyper proliferation. Amplification of type 1 INF production due to genetic material and IL-37 complexation results in the formation of chemokines, which maintains neutrophil infiltration into the epidermis and contributes to psoriasis. Relapses of streptococcal tonsillitis are common, especially in youngsters, and the mechanism is most likely immunologic. People with the condition may also develop a specific type of arthritis that affects the fingers and spine joints. It is unclear if the enhanced rate of keratinocyte proliferation is related to more significant growth-promoting factor activity or the lack of a growth inhibitor.^171^

#### 5.2.1 Pharmacotherapy

Psoriasis treatments try to decrease the proliferation of skin cells and remove scales. Treatment options include topical creams, phototherapy, and oral or injectable medications. The majority of psoriasis patients begin with topical therapy. Dithranol, coal tar, topical corticosteroids, vitamin D3 analogues, calcipotriol, retinoids, and calcineurin inhibitors, among others, are some of the topical medicines used to treat psoriasis.^172−174^ Corticosteroids are the most commonly recommended treatments for mild to severe psoriasis treatment. Salicylic acid shampoos and scalp treatments help to minimize psoriasis scaling on the scalp. The explored research work showed the synthesis of the novel phospholipid loaded with tamoxifen (TAM). The results showed the advantage of TAM-loaded new phospholipid-based systems in treating psoriasis over traditional gels.^88^ The study’s findings were intriguing, as they revealed a high degree of stability, ease of application, and biocompatibility. The results of this study can be applied to enhance the LO systems of various medications, hence improving the safety and efficacy of topical drug administration. A further study investigated the utilization of an organogel formulation containing a topical corticosteroid, fluocinolone acetonide, as a therapeutic regimen for the treatment of psoriasis.^155^ The *ex vivo* drug release lasted for up to 6 h, with a release profile of 90.64%, which was shown to be protracted compared to that of the commercially available preparation. Additionally, a higher cutaneous disposition was observed. Notably, these formulations showed promising results with less frequent dosage, fewer systemic side effects, and better patient compliance.

### 5.3 Cutaneous Infection

Cutaneous fungal infections are the skin, hair, and nail infections that primarily affect the surface tissues. Dermatophytes are the most frequent cause of these fungal infections, but other fungi and yeast (species of *Candida*) can also be responsible.^175,176^ A dermatophyte is a type of fungus that causes tinea, a fungal ailment. As a result, dermatophytosis is referred to as tinea infections, which are further divided according to the area of the body that is infected (for example, tinea pedis and tinea capitis) (Figure 18c).

#### 5.3.1 Pharmacotherapy

Various types of topical therapies, including corticosteroids and antifungals, are available for the treatment of cutaneous infections. These topical therapies are available as ointments, creams, powders and aerosols, which are well tolerated without any skin irritation or inflammation. Clotrimazole, econazole, efficonazole, ketoconazole, sertaconazole, and luliconazole, among others, are the drugs of choice to treat cutaneous infections.^177,178^ Also, oral treatments including griseofulvin, terbinafine, itraconazole, and fluconazole as suspensions or tablets have shown its effectiveness against extensive or severe infections.^179,180^ However, in the reported work on the pluronic lecithin organogel of acyclovir,^157^ the results showed enhanced delivery of acyclovir (ACY) to the deeper targeted site of the skin to treat herpes caused by cutaneous HSV-1 infection. The higher bioavailability and safety observed in the overall performance were attributed due to the synergistic interaction between the properties of excipients and the qualities of the formulation.

### 5.4 Acne

Acne is a follicular skin disorder characterized by inflammatory or noninflammatory lesions and scarring that primarily affects the pilosebaceous unit of the face, neck, and trunk.^181^ Increased sebum production, abnormal keratinization of the pilosebaceous canal, bacterial colonization, and the production of inflammatory factors are all known to play an essential role in the pathogenesis of acne.^182−184^ The most common pathogens involved in this process include *Propionibacterium acnes*, *Staphylococcus aureus*, and *Staphylococcus epidermidis*.^185^ Acne vulgaris typically impacts regions of the dermis that have abundant sebaceous follicles, including the facial area, upper chest, and dorsal region. Acne vulgaris symptoms include discomfort, tenderness, and erythema.^186^ Comedones manifest on the facial region of individuals with a predisposition to acne as a result of an excessive presence of androgen hormones and sebaceous glands in the anterior region of the skin, leading to an increased sebum production. The two noninflammatory lesions in acne are closed comedones (whitehead) and open comedones (blackhead).^187^ When the contents of these lesions rupture, they have the potential to transform into inflammatory papules and pustules. (Figure 18d). It is also possible for larger, more painful cysts and nodules to develop. Novel transdermal delivery systems exhibit gradual dispersion of topical drugs, hence reducing the irritative properties of certain antiacne medications while concurrently demonstrating notable efficacy.^188^

#### 5.4.1 Pharmacotherapy

There are primarily three distinct approaches employed in the treatment of acne:^189^

(a) Topical treatment encompassing the use of antibiotics, retinoids, and other combinations of medicines. Regrettably, a significant proportion of topical acne medications elicit skin irritation.
(b) Systemic treatment encompassing several forms of medication, including oral antibiotics, retinoids, and hormone therapy. Furthermore, it is imperative to address moderate to severe acne by implementing systemic therapy.
(c) In addition to the aforementioned categories, there exist several more therapeutic approaches, namely resurfacing, dermabrasion, chemical peels, xenografts, heterograft, autograft, and fat transplantation, which are distinct from the previously mentioned categories.

**Figure 19:**
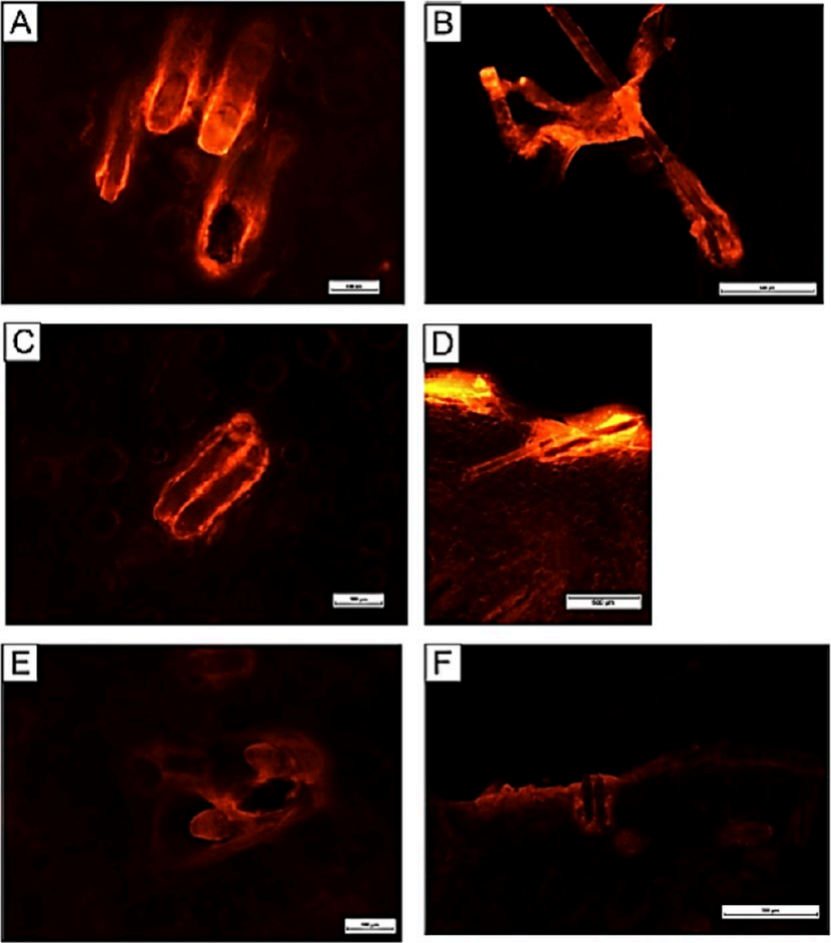
Fluorescence images depicting the distribution of Nile red within hair follicles. The left column displays horizontal sections of the scalp skin, with a depth of approximately 300 μm and an exposure time of 600 ms, after a 5 min penetration period. The right column exhibits vertical sections of the scalp skin, with an exposure time of 1.5 s, following a 1 h penetration period. The experimental setup consisted of two aqueous suspensions, labeled as A and B, containing Nile red/roxithromycin (ROX)-loaded nanoparticles (NPs). Additionally, two pluronic lecithin organogels, labeled as C and D, were prepared, which contained Nile red/ROX-loaded NPs. Lastly, an oily solution of Nile red, labeled as E and F, was also included in the study. Adapted with permission from ref (191). Copyright 2014 Elsevier.

The combination of retinoid and antibiotic therapy for acne has been suggested by the Global Alliance to improve acne treatment outcomes because it is more effective than monotherapy.^190^ An investigation on the delivery of roxithromycin (ROX) loaded nanoparticles from the PLOs to the hair follicles was reported for the treatment of acne.^191^ The results revealed that the ROX was successfully encapsulated into biodegradable, biocompatible, and low-cost polymeric NPs with an approximate size of 300 nm. *Ex vivo* human scalp skin penetration tests demonstrated that polymeric NPs can be used to selectively target the pilosebaceous unit. The particles containing ROX and Nile red showed markedly enhanced follicular penetration behavior in both a water suspension and an organogel compared to an oily solution (Figure 19). In another study, a combination hydrogel and organogel approach was implemented for the formation of bigels containing doxycycline hyclate for the treatment of acne.^192^ The studies showed that the microstructured size of the bigels was within the range of 15–50 mm, which was considered optimal for ensuring stability and uniformity in the formulation. The findings from drug release studies indicated that the bigel formulation had a greater efficacy in terms of achieving a controlled and sustained drug release pattern. This superiority was attributed to the higher concentration of organogel present in the optimized formulation, which contributed to enhanced compatibility and desired drug release outcomes.

## 6 Clinical Relevance of Lecithin Organogels

Subsequent efforts have led to the application of organogels with clinical relevance in various types of diseases conditions. The development of novel polymers, methods for cross-linking polymers, and techniques for fabricating organogels, among others, in the improvement of healthcare are all being facilitated by advancements in these domains. Various organogel-based technologies have received regulatory approval for healthcare applications. To this end, the Diltigesic organogel (2%) is available as a marketed formulation, which is used to reduce pain and promote the healing of a tear that has occurred in the skin of the anus. The main therapeutic molecule present is diltiazem (calcium channel blockers), which is encapsulated in a pluronic lecithin organogel. Table 4 provides the summary of the lecithin-based drug delivery studies in clinical phases.

**Table 4:** Clinical Application of Organogels

sl no.	organogelator used	route of administration	study conducted	model drugs and indications
1	lecithin	transdermal	clinical trials	diclofenac for osteoarthritis^193^
				diclofenac epolamine gel for sprains, strains, and contusions^194^
				diclofenac epolamine gel for shoulder periarthrities and lateral epicondylitis^195^
2	pluronic lecithin	transdermal	clinical trials	12-hydroxystearic acid for human health and nutrition^196^
				silymarin for atopic dermatitis^125^

## 7 Conclusion and Future Perspective

In this review, we have discussed the advancement of organogels with respect to their preparation, characterization, and applications in various skin diseases. Organogels present unique characteristics, including their thermodynamic behavior, viscoelasticity, and versatility. These characteristics can easily be tuned by simple formulation adjustments, resulting in highly structured architectures. Among other organogel-based formulations, LOs have emerged as one of the most promising carrier systems for topical medication delivery. This have also proved to have advantages over other lipid-based systems like vesicular systems (liposomes and niosomes) and semisolid dosage systems in terms of effectiveness, stability, and most importantly technological viability. To this end, most of the lecithin-based organogel formulations are available in the market or at the verge of clinical trials. Permeation and retention of both hydrophobic and hydrophilic drugs across the skin were enhanced, and LOs have significance in the treatment of various skin ailments. LOs have been found to be nonirritating and biocompatible, hence enhancing safety and promoting patient adherence when applied over a long period of time. However, in the future, there is a need to investigate the influence of the organogel components, such as the cosurfactant, organic solvent, or other additives and their concentration, on the kinds of microstructures that are generated within the system, as well as on the topical drug transport mechanism.
